# Supplementary material for: Cerebral cortex activation and functional connectivity during low-load resistance training with blood flow restriction: An fNIRS study
Source: PLoS One. 2024 May 23;19(5):e0303983. doi: 10.1371/journal.pone.0303983 (PMC11115316; doi:10.1371/journal.pone.0303983)
Supplement: S1 Appendix — (DOCX) [file pone.0303983.s001.docx]

**S1 Appendix. Sample Size Estimation**

Based on the 4*3 within-factors design in this study, a repeated measures analysis of variance was selected. The significance level (α) was set at 0.05, and the statistical power (1-β) was set at 0.8. This study examined two effects: the main effect of pressure intensity and the interaction effect of pressure intensity and ROI on cortical activation. Since the main effect of interest had not been previously explored, we referred to the results of a study that used fNIRS to investigate changes in HbO during resistance training under different loads [1]. Subsequently, an effect size f = 0.428 ($\eta_{p}^{2}=0.155$) was chosen to calculate sample size by G*power (as shown in Figure 1), and resulting in N=9. It should be noted that the effect size used here is not the original effect size from the previous study ($\eta_{p}^{2}=0.$31) but a compromise value considering publication bias and statistical error in small-sample studies [2,3]. Regarding the interaction effect, due to the lack of statistical information from similar studies, we used an estimated value $\eta_{p}^{2}=0.1$ to calculate the required sample size and resulting in N = 22. Ultimately, we chose a sample size of N=22 for our study, and the actual number of participants was 24.


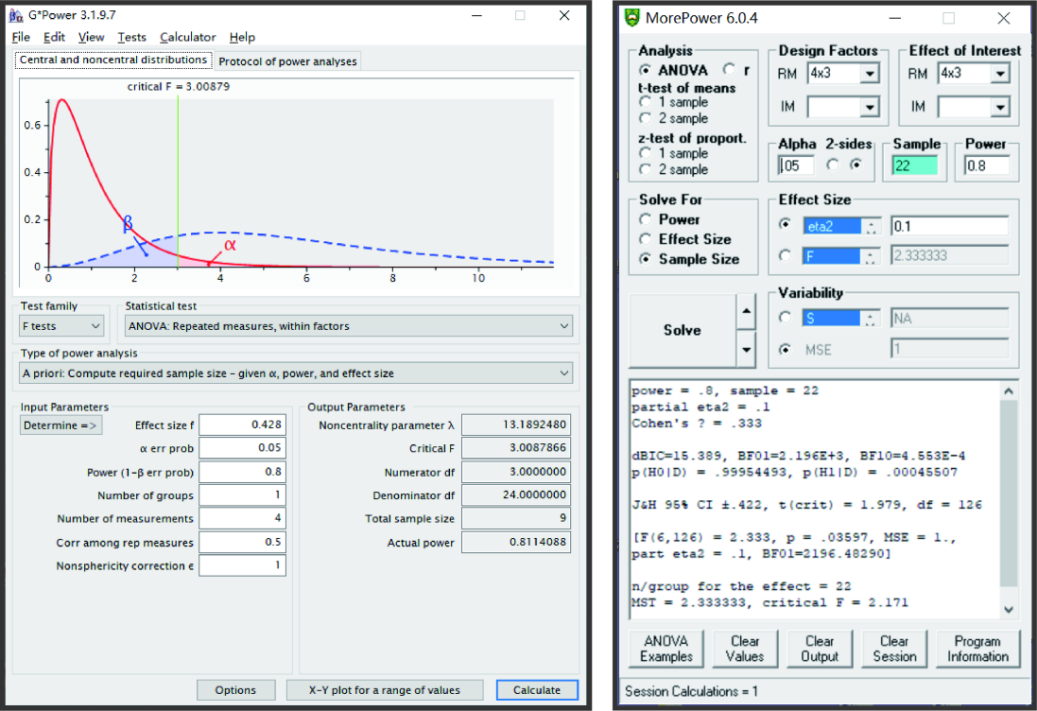


**Figure1. The Prior Power Analysis.** On the left, the sample size calculation for the main effect was conducted using G*Power (<http://www.gpower.hhu.de/>). On the right, the estimation for the interaction effect was performed using MPower (<https://wiki.usask.ca/pages/viewpageattachments.action?pageId=420413544>).

**References**

1. Kenville R, Maudrich T, Carius D, Ragert P. Hemodynamic Response Alterations in Sensorimotor Areas as a Function of Barbell Load Levels during Squatting: An fNIRS Study. Front Hum Neurosci. 2017;11: 241. doi:10.3389/fnhum.2017.00241

2. Open Science Collaboration. PSYCHOLOGY. Estimating the reproducibility of psychological science. Science. 2015;349: aac4716. doi:10.1126/science.aac4716

3. Schäfer T, Schwarz MA. The Meaningfulness of Effect Sizes in Psychological Research: Differences Between Sub-Disciplines and the Impact of Potential Biases. Front Psychol. 2019;10: 813. doi:10.3389/fpsyg.2019.00813
