# Supplementary material for: Cerebral cortex activation and functional connectivity during low-load resistance training with blood flow restriction: An fNIRS study
Source: PLoS One. 2024 May 23;19(5):e0303983. doi: 10.1371/journal.pone.0303983 (PMC11115316; doi:10.1371/journal.pone.0303983)
Supplement: S2 Appendix — (DOCX) [file pone.0303983.s002.docx]

**S2 Appendix. FNIRS Data Process**

Firstly, problematic trials (25 out of 1900 trails, 1.32%,) in which participants failed to complete the squat within the specified time frame during the task were marked and subsequently excluded from further analysis. Secondly, we examined the power spectrum across 22 channels for all participants in Homer3. Channels without clear heart rate components were identified as noisy channels [1]. These noisy channels ( 217 out of 528 channels, 41.1%, as depicted in Figure 2) were removed from data visualization and statistical analysis. Given HbO is more sensitive to cardiac signals compared to HHb, our noise channel identification primarily relied on the power spectrum of HbO [2]. After those pro-processing steps, the participants' raw light intensity signals were transformed into optical density signals by Homer3. Subsequently, Spline interpolation with a Savitzky-Golay filter was used to correct motion artifacts via the "hmrMotionCorrectSplineSG" function in the HOMER3, with parameter settings of p = 0.99, Frame Size_sec=300, and turn on=1. Next, we applied band-pass filtering (0.01-0.2 Hz), using a fourth-order Butter-worth band-pass filter with a finite-impulse response model, to mitigate the influence of low-frequency drift, Mayer waves, respiration, and cardiac components in fNIRS data [3]. Then, the optical density signals were converted into hemoglobin concentration signals ( ppf = 1.0 1.0). Finally, we obtained time-series data for the changes in HbO for each participant under different experimental conditions. Additionally, since the estimation of HbO changes within different regions of interest was primarily based on the specific channels of each brain region, we applied channel-specific weighting to account for differences in sample size and specificity between different channels. The formula for calculating the weight assigned to each channel was as follows: Weight = N_channel_ / N_roi-max_ * S_channel_ / S_roi-max_ (N_channel_: number of participants in the channel, N_roi-max_: maximum number of participants among channels within the targeted ROI, S_channel_: specificity of the channel, S_roi-max_: maximum specificity among channels within the targeted ROI). Lastly, we performed weighted averaging of the data from the top-4 channels within the ROI to obtain HbO mean and statistical analysis across different pressure intensities and ROI during BFR training.


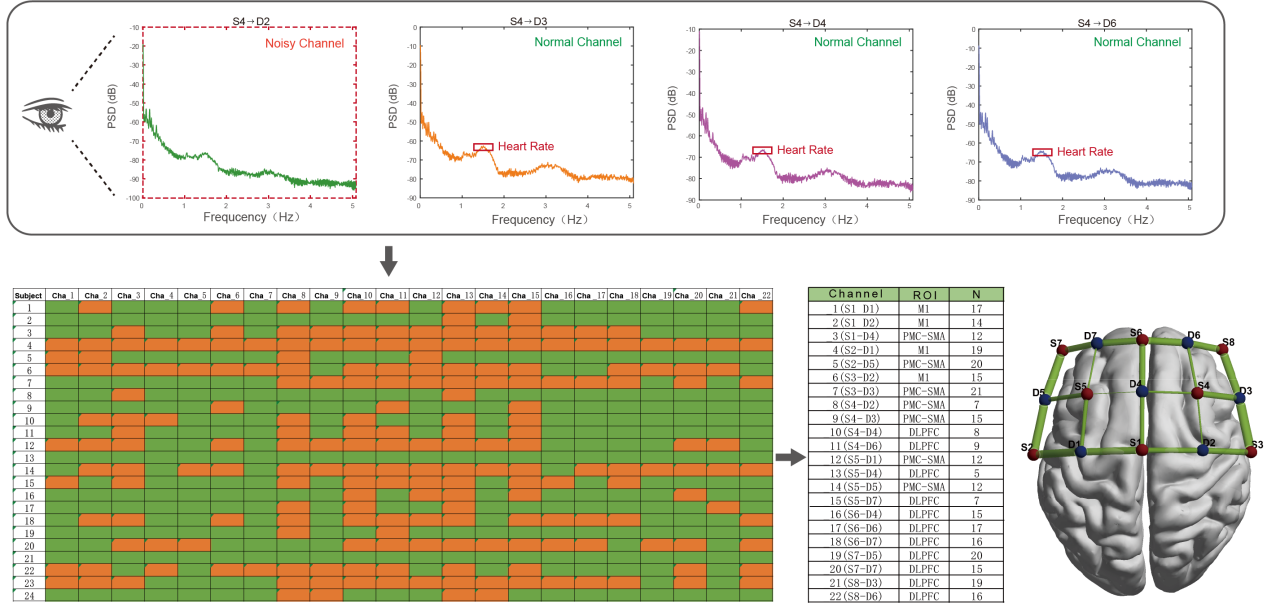


**Figure 2.NIR Data Preprocessing： Noisy Channel identification and data rejection**

**References**

1. Guérin SMR, Vincent MA, Karageorghis CI, Delevoye-Turrell YN. Effects of Motor Tempo on Frontal Brain Activity: An fNIRS Study. NeuroImage. 2021;230: 117597. doi:10.1016/j.neuroimage.2020.117597

2. Pinti P, Scholkmann F, Hamilton A, Burgess P, Tachtsidis I. Current Status and Issues Regarding Pre-processing of fNIRS Neuroimaging Data: An Investigation of Diverse Signal Filtering Methods Within a General Linear Model Framework. Front Hum Neurosci. 2019;12: 505. doi:10.3389/fnhum.2018.00505

3. Yücel MA, Lühmann A v., Scholkmann F, Gervain J, Dan I, Ayaz H, et al. Best practices for fNIRS publications. Neurophotonics. 2021;8. doi:10.1117/1.NPh.8.1.012101
